# Intratesticular Fetus in Fetu Arising from an Intra-Abdominal Undescended Right Testis: A Case Report

**DOI:** 10.70352/scrj.cr.25-0591

**Published:** 2026-02-21

**Authors:** Fuminori Numano, Noboru Oyachi, Satoshi Shinohara, Rei Sunami

**Affiliations:** 1 Department of Pediatric Surgery, Yamanashi Prefectural Central Hospital, Kofu, Yamanashi, Japan; 2 Department of Obstetrics and Gynecology, Yamanashi Prefectural Central Hospital, Kofu, Yamanashi, Japan

**Keywords:** fetus in fetu, testicular origin, undescended testis, unilateral testicular absence, intra-abdominal, neonate

## Abstract

**INTRODUCTION:**

Fetus in fetu (FIF) is a rare congenital anomaly. The most common location of FIF is the retroperitoneum. FIF of testicular origin is extremely rare, with only 9 cases reported previously. We present a case of intratesticular FIF within an intra-abdominal undescended right testis and review previously reported cases to discuss the possibility of intratesticular FIF.

**CASE PRESENTATION:**

A male neonate was born through normal vaginal delivery at 37 weeks and 5 days of gestation, with a birth weight of 2648 g. An abdominal mass suspected to be a FIF was prenatally detected. At 26 weeks of gestation, fetal ultrasonography revealed a mass measuring 20 mm in diameter that contains structures resembling an embryo and an umbilical cord in the abdominal cavity; however, these structures were no longer identifiable after 29 weeks of gestation. Postnatally, a mass measuring 20 mm in diameter was found in the right lower abdomen. The mass was mobile, well defined, heterogeneous, hypovascular, and showed calcifications at its margins. The normal right testis was not identified. The patient’s general condition was stable; therefore, elective laparotomy was performed on the 15th day of life. Intraoperatively, a 20-mm oval mass with a smooth surface was identified in the right lower abdomen. The mass was tethered by a thin fibrous band attached to the right internal inguinal ring, which was presumed to be the feeding vessel. The normal right testis was not observed. Gross examination of the excised specimens revealed massive hematomas and solid structures arranged in a ladder-like configuration, connected to the inner wall of the mass via a cord-like structure. Histopathological examination demonstrated cartilage within the solid structures, which were regularly and segmentally arranged, consistent with vertebral elements. No remnants of testicular, epididymal, or spermatic cord tissue were observed within the mass.

**CONCLUSIONS:**

This case was considered most consistent with FIF arising within an intra-abdominal undescended right testis based on the macroscopic testis-like morphology, absence of a normal right testis, and localization of the lesion in proximity to the normal pathway of testicular descent.

## Abbreviation

FIF
: fetus in fetu

## INTRODUCTION

FIF is a rare congenital anomaly characterized by the presence of a malformed parasitic fetus incorporated into the body of the host fetus. The estimated incidence is approximately 1 in 500000 live births. Most cases arise in the retroperitoneum, and only a few cases have been reported in the testis to date. In this report, we describe a case of intra-abdominal FIF presumed to have originated from the right testis, based on characteristic gross and histopathological findings. In addition, we review previously reported cases to discuss the possibility of intratesticular FIF.

## CASE PRESENTATION

**Fig. 1:**
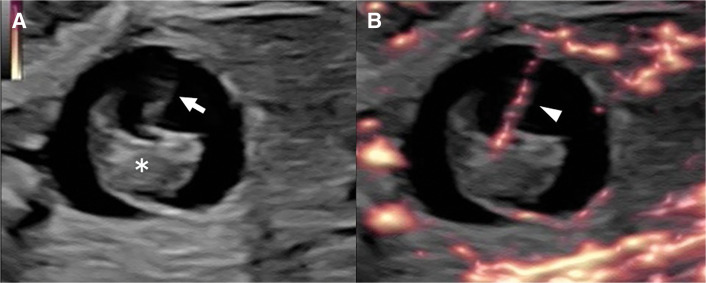
Ultrasound findings at 26 weeks of gestation. (**A**) Cystic mass, 20 mm in diameter, with an internal solid structure (asterisk) and a cord-like structure (arrow). (**B**) Cord demonstrating blood flow (arrowhead). The solid structure resembles an embryo, and the cord-like structure resembles an umbilical cord.

We present the case of a male neonate born at 37 weeks and 5 days of gestation through normal vaginal delivery, with a birth weight of 2648 g. Antenatal sonography at 23 weeks of gestation revealed a 10-mm intra-abdominal cystic mass at the lower pole of the right kidney. At 26 weeks of gestation, the cystic mass enlarged to 20 mm in diameter. Solid components resembling an embryo and a cord-like structure with blood flow resembling an umbilical cord were observed inside the cystic mass; therefore, FIF was suspected (**Fig. 1**). However, by 29 weeks of gestation, these structures had collapsed, and the contents of the mass had become heterogeneous without detectable blood flow. Thereafter, the size and internal structure of the mass remained unchanged until birth.

**Fig. 2:**
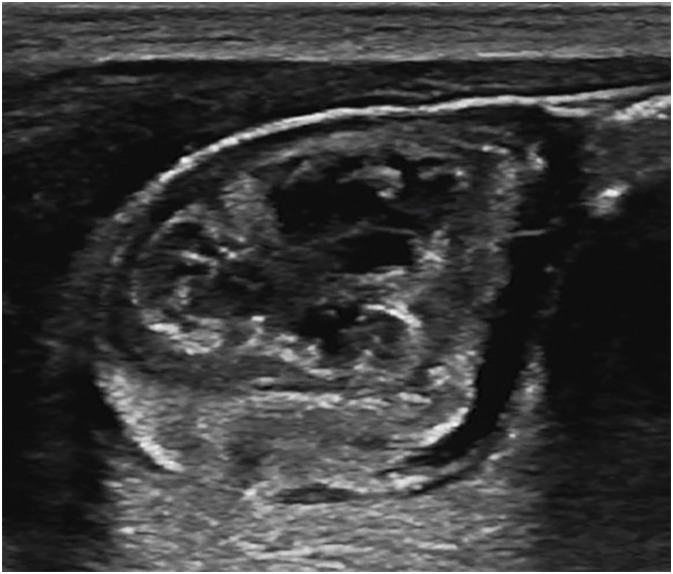
Postnatal ultrasound findings. A 20-mm abdominal mass observed in the right lower abdomen.

**Fig. 3:**
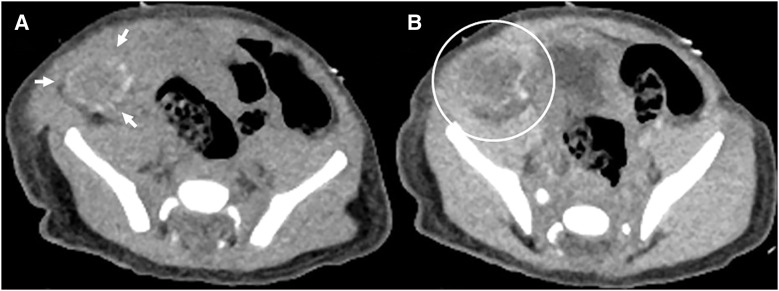
CT findings. (**A**) Plain CT showing a low-density mass with annular calcifications at the margins (arrows). (**B**) Contrast-enhanced CT showing no contrast enhancement in the mass (circle).

Postnatally, ultrasonography demonstrated a 20-mm mass in the right lower abdomen. The mass was mobile, well defined, heterogeneous, and hypovascular (**Fig. 2**). Abdominal CT revealed a low-density mass located on the right side of the bladder, with annular calcifications along its margin. Contrast-enhanced CT showed no enhancement within the mass (**Fig. 3**). MRI demonstrated an organized hematoma within the mass on the cephalic side of the bladder. The left testis was normal in size and morphology; however, the normal right testis was not identified.

**Fig. 4:**
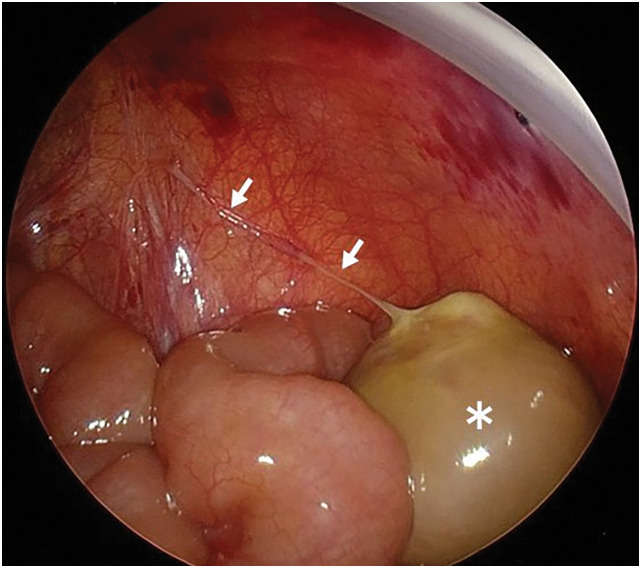
Surgical findings. A 20-mm oval mass with a smooth surface in the right lower abdomen (asterisk) anchored by a single thin fibrous band connected to the right internal inguinal ring (arrows).

The patient’s general condition was stable; therefore, elective laparotomy was performed through a right lower transverse incision on the 15th day of life. Intraoperatively, a 20-mm oval mass with a smooth surface was identified, anchored by a single thin fibrous band. Laparoscopic inspection of the abdominal cavity confirmed that the mass was located in the right lower abdomen and that the fibrous band was connected to the right internal inguinal ring, without any additional adhesions (**Fig. 4**). The mass was detached from its attachment and completely excised.

**Fig. 5:**
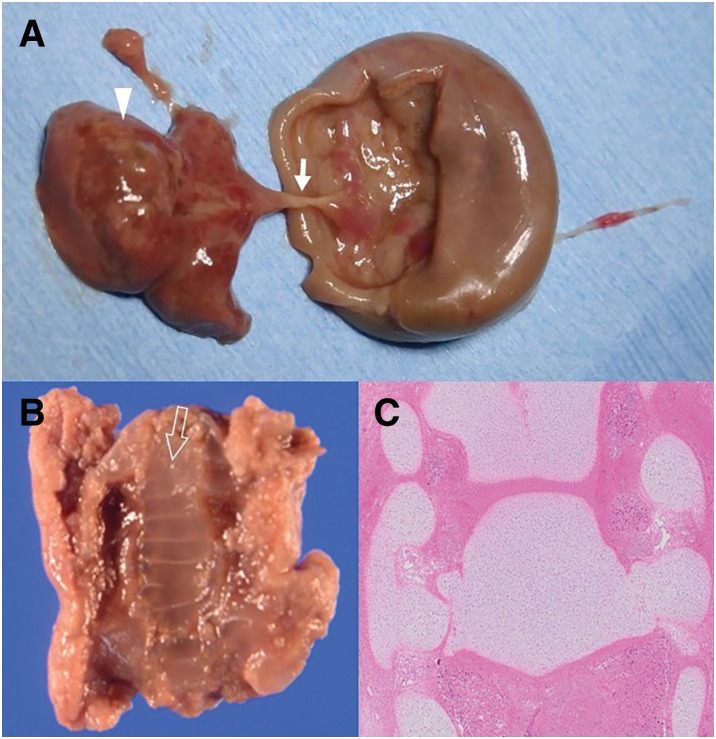
Gross and histopathological findings of the excised specimen. (**A**) Solid structures (arrowhead) connected to the inner wall of the mass through a cord-like structure (solid arrow). (**B**) Solid structures on coronal section, showing a ladder-like arrangement (open arrow). (**C**) Histopathological examination of the coronal section contain cartilage and exhibit a regular and segmental arrangement.

Gross examination of the excised specimen revealed a peach-colored, muddy hematoma and firm, solid structures resembling bone. The solid structures were connected to the inner wall of the mass through a cord-like structure and grossly resembled an embryo and an umbilical cord (**Fig. 5A**). On coronal sectioning, the solid structures were arranged in a ladder-like configuration, resembling a vertebral column (**Fig. 5B**). Histopathological examination demonstrated cartilage within the solid structures, which exhibited a regular, segmental arrangement consistent with vertebral elements (**Fig. 5C**). The cord-like structure was confirmed to be a blood vessel on histopathological examination. Accordingly, the solid and cord-like structures were considered consistent with a fetus and an umbilical cord, respectively. The single thin fibrous band anchoring the mass contained vessels but no vas deferens, and no testicular or epididymal remnants were identified within the mass.

Based on the characteristic gross and histopathological findings and the absence of a normal right testis, the lesion was considered most consistent with FIF arising within an intra-abdominal undescended right testis. Although the fibrous band did not contain a histologically identifiable vas deferens, it was considered as the feeding vessel of the mass and possibly a remnant of the spermatic cord, based on its gross morphology.

## DISCUSSION

FIF is a rare congenital malformation first described by Meckel in the 18th century.^1)^ The most common location of FIF is the retroperitoneum, whereas atypical locations include the intracranial and oral cavities, neck, adrenal gland, liver, scrotum, pelvis, and mediastinum.^2)^ The reported male-to-female ratio is approximately 1.1:1, indicating a slightly higher incidence in males.^3)^ The clinical presentation is nonspecific and depends on the size and location of the lesion. FIF may be detected incidentally or may present with symptoms caused by compression of adjacent structures.^4,5)^ The etiology is believed to involve defective implantation. During the second week of development in multiple pregnancies, one or more embryos may invade the extra-embryonic mesenchyme of the dominant embryo instead of the uterine wall. Following the development of the primitive streaks, as the dominant embryo undergoes delimitation, another embryo may be incorporated within its mesenchyme. This results in parasitic, non-dominant fetuses with vascular connections embedded in various regions of the mesenchymal components of the dominant embryo.^6)^ Currently, no established guidelines exist regarding the treatment or postoperative follow-up of FIF. Although FIF is generally considered benign, complete surgical resection is the recommended treatment, and prognosis is favorable in most cases. However, intracranial FIF grows within the host brain parenchyma, causing severe compression of surrounding brain tissue and accumulation of cerebrospinal fluid, leading to increased intracranial pressure. Consequently, intracranial FIF is associated with a poor prognosis, regardless of whether complete resection is achieved.^7)^ In addition, several reports have described recurrence as teratoma or malignant transformation following incomplete excision.^8–10)^ Therefore, complete excision is recommended; however, careful postoperative surveillance is warranted when complete excision cannot be achieved.^2,4)^

Distinguishing FIF from teratomas is crucial for accurate diagnosis. FIF is widely recognized as a clinical entity distinct from teratomas, and radiographic or pathological evidence of a vertebral structure is considered essential for diagnosis.^11)^ Willis^12)^ and Lord^13)^ argued that FIF is fundamentally distinct from a teratoma. Willis suggested that the diagnosis of FIF should be limited to cases demonstrating a definitive vertebral column, as teratomas lack vertebral structures because they do not pass through the period of primitive streak formation required for vertebral development. Lord further emphasized that a true diagnosis of FIF requires unequivocal radiographic or anatomical demonstration of part or all of the vertebral axis. In contrast, some authors have argued that FIF may represent a highly developed or complex form of teratoma.^14)^ Teratomas, despite containing well-organized structures, are neoplasms originating from embryonic pluripotent cells.^3)^ Several cases have been reported in which FIF was diagnosed based on advanced structural organization despite the absence of a defined vertebral column.^15,16)^ However, these cases are not consistent with the widely accepted clinical entity, and cases lacking vertebral structures remain a matter of debate.

As previously mentioned, FIF can occur in various anatomical locations and may also develop within an intra-abdominal undescended testis. Although intratesticular FIF has been hypothesized to originate from primordial germ cells,^17)^ its etiology remains unclear and could not be definitively determined in the current case. To date, only 9 cases of intratesticular FIF have been identified in a PubMed search, underscoring the rarity of this condition. **Table 1** summarizes the characteristics of all 10 cases,^2,11,17–23)^ including the current case. All reported cases underwent surgical excision and had uneventful prognoses. The intratesticular FIF was located in the scrotum in 5 cases and in the abdominal cavity in the remaining 5 cases. The affected testis replaced by FIF was on the left side in 5 cases and on the right side in the remaining 5. Residual tissue of the testis, epididymis, or spermatic cord was identified in 8 but not in 2 cases (the current case and the case reported by Kakizoe and Tahara^18)^). These findings suggest that the testis may be completely replaced by FIF and that testicular descent from the retroperitoneum to the scrotal sac can occur even in such cases. Taken together, the findings from the current and previously reported cases suggest that intratesticular FIF should be considered when a unilateral testis cannot be identified and FIF is located in proximity to the normal pathway of testicular descent.

**Table 1:** Published cases of intratesticular fetus in fetu (FIF)

Year	Author	Basis for intratesticular FIF		
		Absence of normal unilateral testis	Histopathological remnants (testis/epididymis/spermatic cord)	Presence of FIF in the scrotum
1972	Kakizoe^18)^	Yes; Left	No	Yes
1985	Alpers^19)^	Yes; Left	Yes	No; intra-abdominal
1990	Chateil^17)^	Yes; Right	Yes	No; intra-abdominal
1999	Shin^11)^	Yes; Right	Yes	Yes
2015	Ji^2)^	Yes; Right	Yes	Yes
2016	Landmann^20)^	Yes; Right	Yes	No; intra-abdominal
2016	Khope^21)^	Yes; Left	Yes	Yes
2022	Heitman^22)^	Yes; Left	Yes	Yes
2024	Luo^23)^	Yes; Left	Yes	No; intra-abdominal
2026	Current case	Yes; Right	No	No; intra-abdominal

In the current case, the diagnosis of FIF was not based on the presence of an advanced degree of fetal organization but rather on the identification of a vertebral column. Solid components and a cord-like structure with blood flow observed within the mass at 26 weeks of gestation were subsequently shown on histopathological examination to contain vertebral elements and blood vessels, respectively, suggesting the presence of a parasitic fetus and an umbilical cord. However, these structures were no longer visible after 29 weeks of gestation, presumably reflecting failure of the feeding vessels, leading to hemorrhage, necrosis, and degeneration of the FIF. In addition, the normal right testis could not be identified. The oval mass was located in proximity to the normal pathway of testicular descent, and the fibrous band originating from the internal inguinal ring was considered to represent the feeding vessel of the mass. The gross morphology of the mass and fibrous band resembled that of the testis and the spermatic cord. Nevertheless, histopathological evidence of testicular, epididymal, or spermatic cord tissue could not be demonstrated. The testis is a common site for teratomas, and the etiology of FIF remains under debate; therefore, the possibility that the current case represents a teratoma cannot be completely excluded. However, based on the presence of a vertebral column, the absence of a normal unilateral testis, the location of the mass in proximity to the normal pathway of testicular descent, and the gross morphology of the mass and fibrous band, this case was considered most consistent with an intratesticular FIF arising from an intra-abdominal undescended right testis.

## CONCLUSIONS

We report a case of FIF detected prenatally and successfully resected during the neonatal period, with a favorable outcome. Although the distinction between FIF and teratoma remains a matter of debate, the current case shares key clinical, gross, and histopathological features with previously reported cases. Based on these findings, this case was considered consistent with intratesticular FIF arising from an intra-abdominal undescended right testis.

## DECLARATIONS

### Availability of data and materials

The dataset supporting the conclusions of this article is included within the article.
